# A new method for examining the co-occurrence network of fossil assemblages

**DOI:** 10.1038/s42003-023-05417-6

**Published:** 2023-10-31

**Authors:** Shilong Guo, Wang Ma, Yunyu Tang, Liang Chen, Ying Wang, Yingying Cui, Junhui Liang, Longfeng Li, Jialiang Zhuang, Junjie Gu, Mengfei Li, Hui Fang, Xiaodan Lin, Chungkun Shih, Conrad C. Labandeira, Dong Ren

**Affiliations:** 1 College of Life Sciences, Capital Normal University, Beijing, 100048 PR China; 2 Department of Bioinformatics, Freshwind Biotechnology (Tianjin) Limited Company, Tianjin, 300301 PR China; 3 Beijing Museum of Natural History, Beijing, 100050 PR China; 4 College of Life Sciences, South China Normal University, Guangzhou, 510631 PR China; 5 Tianjin Natural History Museum, Tianjin, 300203 PR China; 6 Institute of Vertebrate Paleontology, College of Life Science and Technology, Gansu Agricultural University, Lanzhou, 730070 PR China; 7 College of Agronomy, Sichuan Agricultural University, Chengdu, Sichuan 611130 PR China; 8 Institute of Paleontology, Hebei GEO University, Shijiazhuang, 050031 PR China; 9 Key Laboratory of Green Prevention and Control of Tropical Plant Diseases and Pests, Ministry of Education, School of Plant Protection, Hainan University, Haikou, 570228 PR China; 10 Department of Paleobiology, National Museum of Natural History, Smithsonian Institution, Washington, DC 20013-7012 USA; 11 Department of Entomology, University of Maryland, College Park, MD 20742 USA

**Keywords:** Palaeoecology, Ecosystem ecology

## Abstract

Currently, studies of ancient faunal community networks have been based mostly on uniformitarian and functional morphological evidence. As an important source of data, taphonomic evidence offers the opportunity to provide a broader scope for understanding palaeoecology. However, palaeoecological research methods based on taphonomic evidence are relatively rare, especially for body fossils in lacustrine sediments. Such fossil communities are not only affected by complex transportation and selective destruction in the sedimentation process, they also are strongly affected by time averaging. Historically, it has been believed that it is difficult to study lacustrine entombed fauna by a small-scale quadrat survey. Herein, we developed a software, the *TaphonomeAnalyst*, to study the associational network of lacustrine entombed fauna, or taphocoenosis. *TaphonomeAnalyst* allows researchers to easily perform exploratory analyses on common abundance profiles from taphocoenosis data. The dataset for these investigations resulted from fieldwork of the latest Middle Jurassic Jiulongshan Formation near Daohugou Village, in Ningcheng County of Inner Mongolia, China, spotlighting the core assemblage of the Yanliao Fauna. Our data included 27,000 fossil specimens of animals from this deposit, the Yanliao Fauna, whose analyses reveal sedimentary environments, taphonomic conditions, and co-occurrence networks of this highly studied assemblage, providing empirically robust and statistically significant evidence for multiple Yanliao habitats.

## Abstract (web summary)

A new method and its companion software to study the associational network of lacustrine entombed fauna, or taphocoenosis. The application allows researchers to easily perform exploratory analyses on common abundance profiles from taphocoenosis data.

## Introduction

Compared with modern ecology, palaeoecology clearly encounters greater difficulties in obtaining high-quality samples and derivative data. This situation is largely attributable to palaeoecological research that relies on organisms occurring in ancient environments that have been preserved for long intervals of time. Each development in fossil formation prior to eventual anthropogenic discovery is the result of considerable loss and change in information associated with the original community^1^. Taphonomic data are often seen as a hindrance to correct for rather than provide actual information^2,3^, but some pioneering studies in freshwater/terrestrial environments have demonstrated the use of taphonomy to derive environmental information and it would be worth to highlight at least some of them^2–4^. Taphonomic processes have long been neglected in ecological studies of fossil communities, especially in the freshwater/terrestrial realm, even though evidence from taphonomy can provide important contributions toward interpreting past relationships among organisms, the biological community that they interacted with, the environment they inhabited, and at a much larger scale, the evolution of ecosystems^2,5,6^.

The impact of preburial and postburial taphonomic processes on a once live community of interacting organisms (a biocoenosis) consists principally of two major aspects. First, time drives the incompleteness of the original community^7,8^. Compared to a biocoenosis, a death assemblage of organisms (a thanatocoenosis), consists of a relative abundance of organisms that is severely distorted by selective representation in time, physical destruction and geochemical alteration of the remains^9,10^. Factors such as membership in a taxonomic group, body size, presence of skeletal elements, and depositional environment determine if and how complete biological remains become preserved in the fossil record.

A second issue is that palaeoecological research understandably has a poor assessment of the time interval during which the burial of organisms occurs. Most fossil assemblages are affected by time-averaging due to the mixing of remains of communities^10,11^. Consequently, fossils occurring in the same stratum might have died and been buried at different times. The significance of a taphocoenosis influenced by time-averaging is still controversial in ecology, but the mainstream viewpoint is a taphocoenosis can reflect a community under average environmental conditions, or certainly constitute the taphonomically altered sum of a community in a particular habitat over some interval of time^9^.Third, there also is space averaging, in which skeletal elements are transported to sites of deposition that are distant from their original sources^8^. Factors leading to time and space averaging involve biological, sedimentary, physical, and chemical diagenetic processes caused by a plethora of activities ranging from disaggregation of carcasses from predators, scavengers and microorganisms, transportation of skeletal elements by river currents, exhumation and redeposition by erosive and sedimentary processes, and diagenetic alteration by mineral-laden groundwater^7,11–16^. Nevertheless, time and space averaging can be an advantage, reflecting the average complement of organisms over a relatively narrow interval of time in a commensurately confined space.

**Fig. 1:**
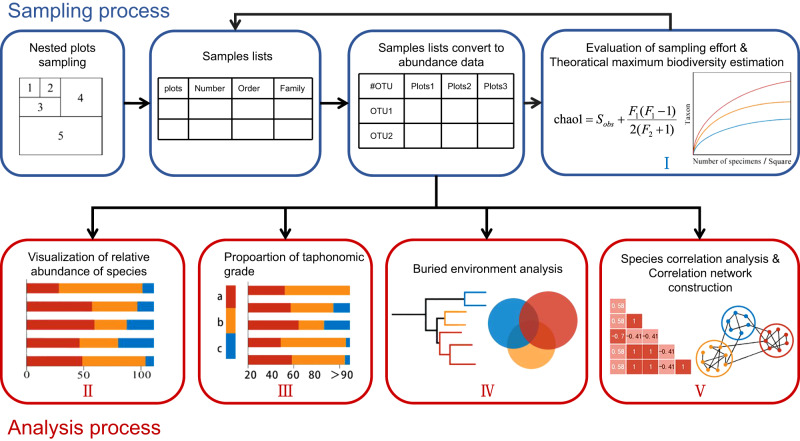
The main functions of *TaphocoenosisAnalyst* software. The data source entered by the user is a sample table, and the detailed format is shown in Supplementary Data 1. The software automatically converts the sample table into an OTU abundance information. Function I draw sampling coverage curves to evaluate sampling depth. Subsequently, the software enters the visualization stage, where all the data analyzed comes from the OTU abundance and taphonomic preservational grade. Visualization of OTU abundance ratios in different plots using Function II. Function III visualizes the proportion of different plots and OTU taphonomic preservational grade. Function IV can cluster sedimentary environments based on the relative abundance of aquatic organisms in different areas.

Although distorted by a variety of processes, taphonomic evidence can reflect the symbiotic relationships of taxa to a certain extent^16–18^. However, studies using network-based methods almost always have been based on large-scale marine faunistic studies, resulting in the virtual absence of smaller scale faunistic studies such as those in lacustrine environments in which presence/absence data in previous studies could not show changes in abundance^16–18^. Therefore, we developed a new method and companion software to study the assemblage network of an entombed fauna, or taphocoenosis (Fig. 1).

## Results

**Fig. 2:**
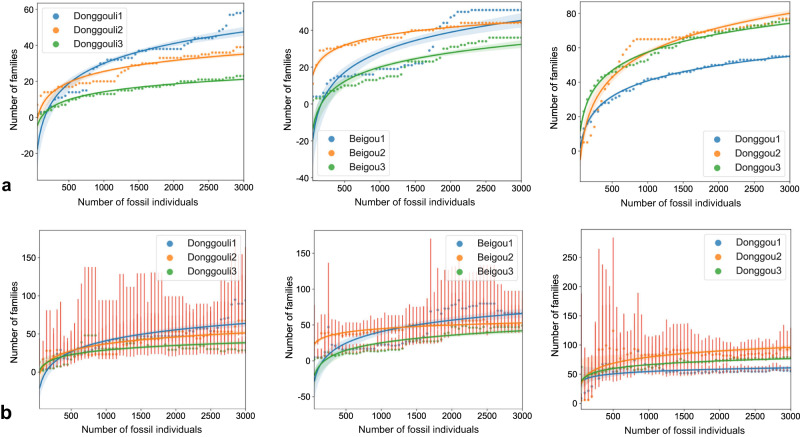
Sparse sampling. **a** Sampling coverage curves (S_obs_). **b** Potential diversity curves calculated based on the Chao1 estimator.

We fitted the sampling depth curve to the number of individuals based on the Chao1 and S_obs_ estimators for all sampling plots. The sampling curve calculates the diversity change every 50 samples and is fitted as a logarithmic function. The sampling depth-curve fit is in the form of a logarithmic function (Fig. 2a). The S_obs_ curve rises rapidly when the number of samples is less than 500 and tends to be flat when the number of samples is more than 1000. When the sampling depth of all quadrats reaches about 3000 samples, the slopes of S_obs_ curve are 0.002092–0.005316 (Table 1, Fig. 2a), which indicates that the sampling is sufficient. At the family level with the S_obs_ estimator at 3000 samples, the S_obs_ curve of Donggouli and Beiguou are lower, having only 21.1–47.5 families with the number of samples at 3000. In contrast, Donggou has 55.1 to 80.1 families with the number of samples at 3000 (Fig. 2a). The Chao1 curve was flat when there were about 500 samples. When the sampling depth of all quadrats reaches about 3000, the slopes of Chao1 curve are 0.001926–0.006907 (Table 1, Fig. 2b). At 3000 samples, Donggouli and Beiguou are also lower in Chao1 curve, with Bonggouli represented by only 41.9–66.0 families and Donggou with 61.0 to 96.0 families (Fig. 2b). The ratio between S_obs_ and Chao1 is 0.55 for Donggouli3, but for the rest it is 0.68–0.97 (Fig. 2).

**Table 1:** Sampling curve slope (*n* = 3000).

	S_obs_	Chao1
Donggouli1	0.005316	0006907
Donggouli2	0.002914	0.00438
Donggouli3	0.002092	0.002912
Beigou1	0.005242	0.007699
Beigou2	0002341	0.002429
Beigou3	0.003738	0.005088
Donggou1	0.00434	0.005088
Donggou2	0006932	0.005057
Donggou3	0005104	0.003133

**Fig. 3:**
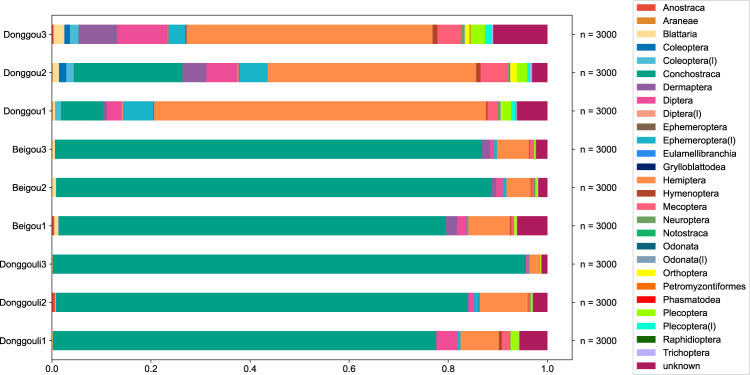
Compositional proportion of the Yanliao Fauna by taxa from the sampled plots. Taxon level is order.

The sample sets of the three localities consist of 27,000 total animal specimens, of which 25,796 (95.5%) specimens are identified to the taxonomic order level (Supplementary Data 1). The hydrophytic (aquatic) assemblage exhibited low diversity and high abundance. The three dominant hydrophytic species are the clam shrimp (conchostracan) *Triglypta haifanggouensis* Liao, Shen & Huang, 2017 (Triglyptidae), water boatman *Yanliaocorixa chinensis* Lin, 1976 (Corixidae), and mayfly *Mesobaetis sibirica* Brauer, Redtenbacher & Ganglbauer, 1889 (Mesonetidae)^19–21^. *Triglypta haifanggouensis* occupies a very high percentage (77.2–95.4%) of the abundance from taphocoenoses at Donggouli and Beigou. In the Donggou plots, aquatic associations were dominated by *T. haifanggouensis*, *Y. chinensis*, and *M. sibirica*, accounting for, respectively, 0–21.9%, 34.8–63.2%, and 2.2–4% of the abundances (Supplementary Data 1). However, terrestrial assemblages often have unstable abundances and are elevated in diversity (Wang et al., 2019). Diptera (true flies), Mecoptera (scorpionflies) and Plecoptera (stoneflies) account for most of the abundance in plots where habitats are adjacent to water bodies, settings favorable for the formation of fossils. The numbers of Dermaptera (earwigs) and Blattaria (cockroaches) also are high, likely attributable to toughened, leathery tissues forming the body surfaces and wings of these insects (Fig. 3).

**Fig. 4:**
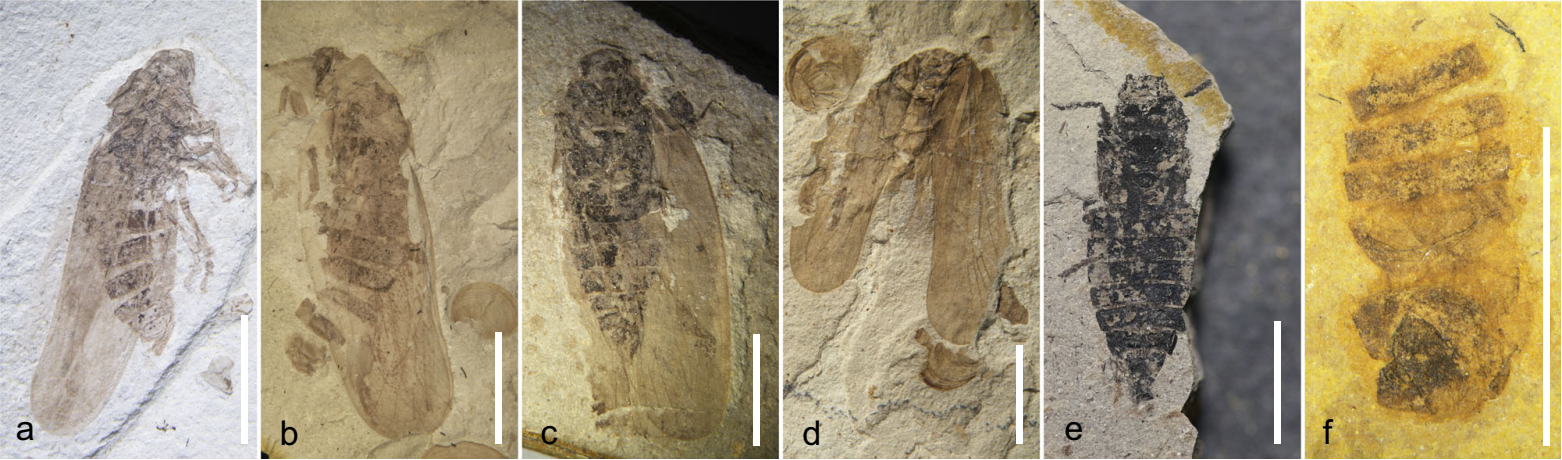
The standard taphonomic grades of Procercopidae. **a** Grade A; **b** Grade B; **c** grade C; **d** grade D. **e**, **f** Grade E. All scale bars are 5 mm.

**Fig. 5:**
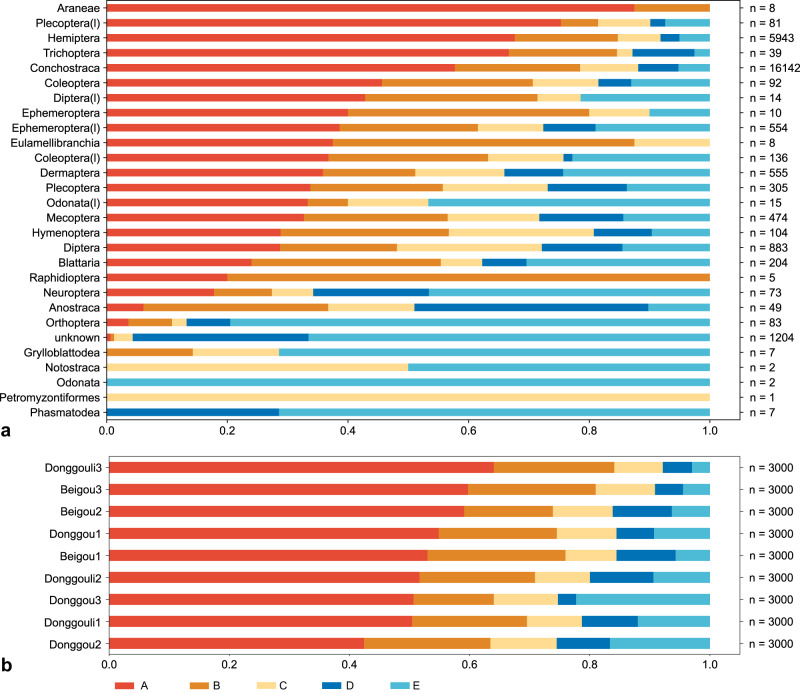
Proportion of taphonomic grades. **a** Proportion of taphonomic grades (A–E) of the Yanliao Fauna by taxa. **b** Proportion of taphonomic grades (A–E) by sampling plot.

From an inventory of 27,000 individual fossils, we found that aquatic organisms are well-preserved, mostly at a high level of body completeness. Although it cannot be determined whether most *T. haifanggouensis* are articulated due to their burial position, nevertheless 78.4% are observed to have a complete chitinous shell and evident growth bands. In addition, >87.8% of *Y. chinensis* are well-preserved, displaying entire bodies and swimmeret appendages while 79.6% of mayfly nymphs have preserved gills and cerci. Furthermore, beetles that have hardened wing covers, true bugs with leathery tegmina, and cockroaches and earwigs that have highly keratinized bodies also are preferentially preserved. By contrast, insects with softer body surfaces and more delicate wings, such as mosquitoes, stoneflies, mayflies, and katydids, are less well-preserved (Supplementary Data 1, Figs. 4 and 5a). Generally, the distance between the insect-occupying habitat and a water body of eventual burial is one of several decisive factors in the preservation of such insects^8^. Trichoptera adults (84.6%), Ephemeroptera adults (80.0%), and Plecoptera adults (48.1%), whose larvae and naiads live in water bodies, have preservation grades of A or B. Mecoptera, Hymenoptera (sawflies and wasps), Diptera, and Neuroptera (lacewings), which live in humid environments, have 56.5%, 56.7%, 48.1%, and 27.3% scores, respectively, at A or B grades. Alternatively, Orthoptera (grasshoppers and katydids), Phasmatodea (stick insects), and Grylloblattodea (ice crawlers) have the least degree of preservation, consisting of 0–10% scores at A or B grades (Table 2, Figs. 4 and 5a). By comparing the taphonomic grades from the nine plots, we found that the differences were small. About 70% of the individuals are preserved at the A and B grades. These observations show that most fossils are buried in an autochthonous or sub-autochthonous manner, which is an ideal place for sampling (Fig. 4b).

**Table 2:** The standard of taphonomic grades.

Taphonomic grades	Preservation		
	Clam shrimps and bivalves	Other arthropods	Vertebrates
A	Shell edge >90% preserved. growth bands are fully clear.	>90% preserved. Body articulated, wing veins visible and almost complete.	Body and limbs are complete and articulated.
B	Shell edge >70% preserved. growth bands are almost clear.	80–90% preserved. Body almost complete, including head, thorax, abdomen and thoracic appendages, details such as antennae or cerci lost.	70–80% torso and limbs are complete. Partial joint displacement.
C	Shell edge >60% preserved. growth bands are partially clear.	60–80% preserved. Body deformed, at least one of six legs lost.	60–70% torso preserved.
D	Shell edge >50% preserved.	30–60% preserved. Wings disarticulated, remains of head, thorax and abdomen preserved.	Torso with missing tail or head.
E	Shell fragments.	<30% preserved. High disarticulated body, isolated structures such as single legs, abdomen and/or wings preserved.	Scattered bones.

**Fig. 6:**
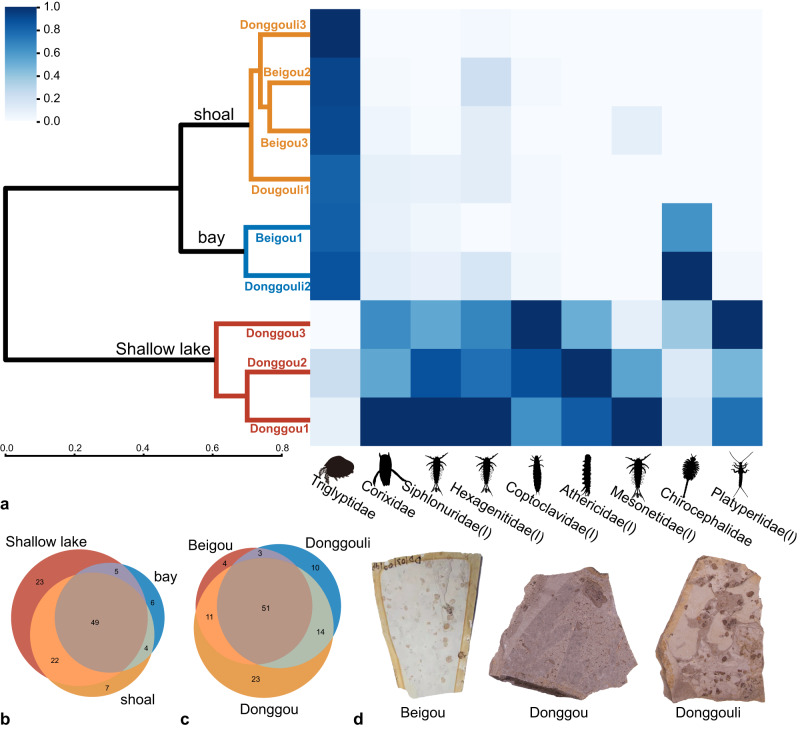
Diversity and abundance comparisons of different plots and sedimentary environments. **a** Hierarchical clustering of nine plots of sedimentary environments. Plots were clustered based on aquatic taxonomic abundance (*n* > 5) using the Average assigns (clustering methods) and Bray-Curtis distance. The clustering results show that the sedimentary environments can be divided into bay, shoal and shallow lake. **b** Venn diagram displaying taxonomic overlap of sedimentary environments. **c** Venn diagrams showing taxonomic overlap of sampling locations. **d** Typical lithologies of the shoal, bay, and shallow lake environments.

The results of a hierarchical cluster analysis within the nine sampling plots indicate that the biofacies of the Yanliao Fauna can be divided into three sedimentary environments (Fig. 6a). The cluster analysis of biological abundance data produced results that paralleled the same three sedimentary environments based on lithology. We find that the dominant taxa in the hydrophytic facies, such as Triglyptidae, Corixidae, and Mesonetidae and other mayfly families were distributed along a lake depth gradient ranging from shallow to deep. Based on the abundance of aquatic OTUs (Operational Taxonomic Units) in different plots and their differences in the occupation of optimal water depth and sedimentary facies among the three groups of dominant species, we assigned the taphonomic environments to shoal, bay, and shallow lake, respectively (Fig. 6).

Based on randomization tests, this threefold community structure is statistically significant (Pearson correlation > 0, *P* < 0.1). Colors were used to designate modules identified using the Louvain Community-Detection algorithm. This algorithm segregates families into five modules (Fig. 6). The first is an aquatic assemblage (orange) that includes water boatman (Corixidae); mayfly naiads of Mesonetidae, Siphlonuridae, and Hexagenitidae, naiads of stoneflies and dragonflies, *Daohugounectes primitinus* Wang, Ponomarenko & Zhang, 2009, *Ferganoconcha sibirica* Chernyshev, 1937 and the lamprey Petromyzontidae gen. sp. 1^22,23^. This assemblage represents a deep-water environment. Second, is a mudflat assemblage (blue), that includes adults of soldier flies (Kovalevisargidae), axymyiids (Axymyiidae), snipe flies (Rhagionidae), primitive crane flies (Tanyderidae), sinoalids (Sinoalidae); and adults of aquatic insects such as primitive minnow mayflies (Siphlonuridae) and rolled-winged stoneflies (Leuctridae). This assemblage is typical of wet environments colonized by herbaceous plants adjacent to water bodies. Third, is the edaphic assemblage (red) that consists generally of cockroaches of the Fuziidae and Blattulidae; earwigs of the Bellodermatidae, Dermapteridae, and Turanodermatidae; and a beetle assigned to Lasiosynidae. This assemblage expressed an affinity for an above-ground environment of humus and litter of ambient vegetation. Fourth, is the silvan assemblage (bright green) that includes a green lacewing (Chrysopidae), a wood gnat (Rhyphidae), an antlion (Myrmeleontidae), a lacewing (Grammolingiidae), and a primitive, cicada-like form (Protopsyllidiidae). This assemblage shows a preference for a relatively dry environment. A small assemblage (grey), including scarab beetles (Scarabaeidae), caddisflies (Economidae), and primitive crane flies (Eoptychopteridae), probably is present that inhabited forests. Notably, the clam shrimp *T. haifannggouensis* has an outsized abundance in the plots but does not appear in the network. For some Branchiopoda species, pH of 5–6 and high elevated free pCO_2_ are the hatching stimuli^24^. In most cases, low oxygen tensions inhibited or strongly reduced hatching^25^. Hypoxia above a certain threshold level, generally no further influence could be detected^24^. Some of these Daohugou layers had highly elevated densities of clam shrimps, with an extreme case of 3000 per square meter. This cluster distribution would explain Daohugou water environment probably in a relatively hypoxic state^24,26^. The absence of fish in Daohugou and the presence of salamanders may support this point for salamanders having lungs to assist in breathing.

**Fig. 7:**
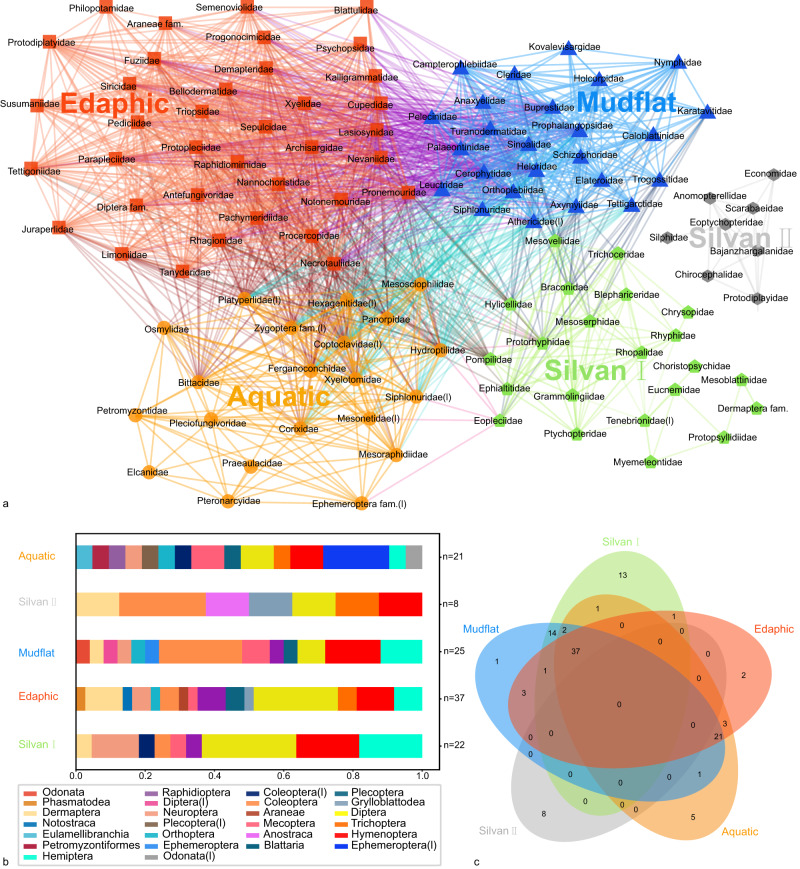
The network and diversity of co-occurrences in the Yanliao Fauna at Daohugou. **a** Co-occurrence networks using the Louvain algorithm. Two families are linked if they have a taphonomic correlation. (Pearson correlation > 0, *P* < 0.1). All families fall into five modules: Aquatic (orange), Mudflat (blue), Edaphic (red), Silvan (bright green and grey). **b** Abundance of the different microenvironments in family level diversity. **c** Family level diversity of the different microenvironments.

**Fig. 8:**
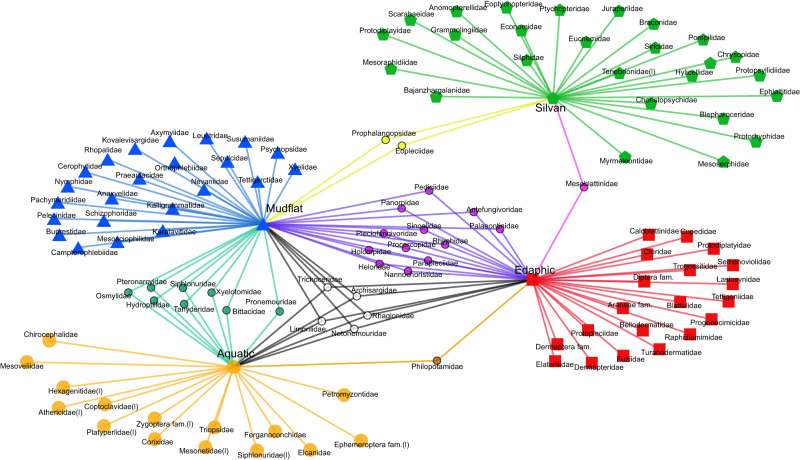
Bipartite network of paleoenvironments and the Yanliao fauna at Daohugou. The data sources are morphologic functions and taxonomic uniformitarianism evidence. Families are linked if they live environments that represent some or all of their same life history. Visualize networks in Python using functions from the following libraries: networkx and matplotlib.

In order to study the ecological significance of taphonomic co-occurrence networks, we compared coexisting networks with traditional networks based on morphological function and taxonomic uniformitarianism. Previous studies of the Yanliao Fauna classified species into Silvan (Woodland), Edaphic (Soil/litter), Aquatic (Hydrophytic), and Montane (Upland forest) assemblages based on a taxonomic uniformitarian approach^27,28^. Because the taphonomic co-occurrence networks are difficult to reflect alpine organisms, and the taxonomic uniformitarian network does not distinguish between shoals and forests, we have made modifications to the previous taxonomic uniformitarian network so that it can be compared with the taphonomic co-occurrence network. Thus, 75.2% of the nodes were correctly assigned in the taphonomic co-occurrence networks (Figs. 7 and 8). Taxonomic uniformitarianism may be misleading for taxa which may have substantially changed their habitats in more recent geological time without obvious morphological change. However, aquatic/terrestrial life habits are generally more conservative and generally less variable. Consequently, 93.8% of nodes were correctly assigned to aquatic/terrestrial life habits in taphonomic co-occurrence networks (Figs. 7 and 8, Supplementary Table 1).

## Discussion

In this study, we developed a research paradigm to reconstruct the latest Middle Jurassic Yanliao communities using taphonomic evidence. Based on the sample sets of 27,000 total animal specimens in three localities, we demonstrated that under less-than-ideal circumstances, such as time and space averaging, and varied transportation modes of organisms to their eventual resting place, that a specific taphocoenosis can still retain a large amount of community-level information. With appropriate sampling methods, our analytical methods show great potential for inferring the ecology of ancient communities.

We entertained additional questions about the aquatic environments in the Yanliao Biota. Our cluster analysis results show that the burial environment at Daohugou can be divided into shoal, bay, and shallow lake. As the water depth increases, the abundance of mayflies and water boatmen gradually increases. Mayfly larvae use gills for respiration, which indicates their occurrence in the benthos and at particular water depths. Based on the depths of water bodies where analogous extant mayfly naiads inhabit, the bathymetry of the three sampling locations did not exceed one meter. We still don’t know whether deeper water environments and associated biological organisms existed. The water environment of Yanliao Biota at Daohugou was quite different from modern water environments. Curiously, bony fish fossils have not been found at the Daohugou locality, and the ecological niches accommodating aquatic, secondary consumers might have been replaced by taxa such as salamanders^29^. However, in the Donggou samples, a lamprey was found that may indicate there was a hidden and complex underwater world within the Yanliao Biota (Supplementary Fig 1).

There are two types of errors in this network: module-defined errors and misconnections. Module-defined errors are attributable to the universality and complexity of biological links (Figs. 7 and 8, Supplementary Table 1). The Mudflat assemblage and the Edaphic assemblage have certain module-defined errors, which are due to the overlapping habitats of the two groups (Figs. 7 and 8, Supplementary Table 1). Additionally, a few rare species clearly are misconnected in the network, especially certain terrestrial taxa that are linked to the aquatic module. Many taxa may not be found in all nine sampling plots because of their low abundance (Figs. 7, and 8, Supplementary Table 1). It is difficult to distinguish whether a zero value in abundance represents a true absence or a presence below the detection level^30^. Zero-abundance matching of the two taxa provides a strong correlation of two species^30^. The simultaneous zero abundance of two OTUs in the plots is not evidence of a positive correlation, because the causes of the zero abundance may be completely different. Zero-abundance matching may be caused by the lack of an overlap between activity periods and rare species abundance below the detection level^30^. Taphonomic correlation may be misleading for the actual abundance changes under the detection level. Consequently, it may be a worth try in future research to filter the abundance data if the abundance of rare species is too small, and their position in the ecological network is significantly affected by randomness^31,32^. However, the commonly used method such as filtering out OTUs with a relative abundance less than 0.1% in the microbiome may not be suitable for direct use in palaeoecological research because it may lead to filtering out most alpine species, large predators, and rare species^31,32^. In future research, we will refer to more additional data-filtering methods from microbiome analyses.

There are a variety of fossil preservation modes in Daohugou biota, which indicates that there were different microenvironments and different fossilization processes in the lake systems at that time^33^. However, more detailed studies are needed on how various microenvironments affect fossil formation^33^. Nevertheless, data biases can be significant. Such biases are mainly due to the degree of keratinization of the body and the difficulty in identification. For example, the abundance of katydids and cockroaches in a community is amplified by their scattered wings with high keratinization and their easy identification.

According to the list of insects described in the Yanliao Fauna, there are 191 families and only 58.1% of the families are found in the sampling process^27,34^. This situation is likely caused by two reasons. First, the Yanliao communities have high patchiness. Such spatial heterogeneity is the principal reason for accommodating high biodiversity. Second, species succession during the deposition of sediment should also be considered, in which the initial cohort of species can be different from the terminal cohort of species when deposition ceases. For the Yanliao Fauna, the depositional interval of time span is 164–157 Ma, during which sufficient time had elapsed to replace many rare species^35^. In general, there are reasons to believe that Yanliao Fauna is a Ship of Theseus phenomenon. Ship of Theseus is a tory and in the way of things with parts needing constant replacement. In the process of constantly changing parts, the renewed Ship of Theseus drives into a paradox whether it is the same ship? In such a community that had many or most of its original species replaced but represented no significant changes in the characteristic species and overall appearance.

Based on the principle of taxonomic uniformitarianism, the Yanliao Fauna is inferred to have had a complex diversity along a vertical gradient. Some taxa are considered alpine or boreal organisms, such as Grylloblattodea, Raphidioptera (snakeflies), some Ephemeroptera, Plecoptera, Prophalangopsidae (grigs), and Osmylidae (giant lacewings) (Supplementary Fig 2)^28,34^. But whether these taxa were montane organisms during the Middle Jurassic is controversial due to the absence of morphological evidence. In terms of abundance and preservation, montane species should be relatively rare and poorly preserved because they must be transported postmortem to a lake by running water. *Mesobaetis sibirica* (Siphlonuridae), *Shantous lacustris* (Hexagenitidae) and *Pronemoura* sp. 1 (Pronemouridae), the most common aquatic insects in the Yanliao Fauna, are considered as montane taxa in previous studies^28^. Extant mayfly and stonefly nymphs occupy streams and rivers, but they can also occur in standing water bodies such as lakes and ponds^36,37^. Characterized by their abundant preservation as fossils, we regard *M. sibirica*, *S. lacustris* and *Pronemoura* sp. as principally lacustrine species living in low altitudes. Nevertheless, it is unknown whether other species were present from upland habitats such as alpine zones.

There is clearly room for improvements in the *TaphonomeAnalyst* approach, as it has only provided networks from taphonomic correlations. To fully understand biological behaviors and interactions, more than one type of ecological data should be collected, integrated, and analyzed. Future development of *TaphonomeAnalyst* will be devoted to a more integrated analysis of different types of palaeoecological data, such as functional morphology, damage types, sporopollen taxa, and body size^38^. The goal of the *TaphonomeAnalyst* is to construct multilayer ecological networks by convenient use of a variety of fossil community-level data.

## Methods

### Fossil sample collection and identifications

Our fossil sample sets were collected from the latest Middle Jurassic Jiulongshan Formation at Daohugou Village, in Ningcheng County of Inner Mongolia, China (Supplementary Fig 3). Based on the stratigraphic interfingering of a lacustrine and a volcaniclastic apron facies, the sedimentary environment of Jiulongshan Formation is interpreted as several fluctuating, shallow lakes with distant volcanogenic sedimentary input^34,39^. Radiometric evidence shows that the depositional age of the Jiulongshan Formation is 165–164 Ma and is time equivalent to the Haifanggou Formation in Liaoning Province^39^. The Daohugou assemblage of organisms, known as the Yanliao Biota, has attracted a great deal of scholarly attention due to issues related to the potential origin of angiosperms, pollinating and blood-sucking insects, feathered dinosaurs and early mammals^29,40–50^. The diversity of vertebrates documented in the Yanliao Fauna is relatively low, with about 20 species known to date^29^. Currently, salamanders, dinosaurs, pterosaurs, and mammaliaformes have been documented, but curiously, fish have not been reported^28^. The invertebrates of the Yanliao Fauna are principally clam shrimp, bivalves, insects, and spiders^19,28,29,34,51–53^. As an important sign of the Yanliao Fauna, the clam shrimp *Triglypta haifanggouensis*, is often densely distributed in the shoal sedimentary facies and is the most abundant organism of the taphocoenosis^28^. Insects are the most diverse taxa in the Yanliao Fauna, and they have important roles of the local community. More than 900 insect species have been described since the Yanliao insect fauna was initially reported in 1983 by Hong^27,34,50^.

The fossils of the Daohugou locality contain well-preserved body structures such as evident tentacles, mouthparts, and wing veins^27,28,34,50^. Based on careful examination of the strata, fossil preservation, and sedimentary environmental context, it can be inferred that a shallow lake was present which lacked strong currents and other factors that would interfere with typical lacustrine deposition. There are several fossil preservation types at Daohugou, which indicate that different subenvironments were present in the lake system at that time. We selected three fossil locations for excavation based on lithology (Fig. 6d). The center of each sampling plot was separated 2 m apart from an adjacent sampling plot (Supplementary Fig 3). The lithologies of all strata from the three locations consist of well-bedded, tuffaceous shale.

In our example dataset, the horizontal deviation in a sampling plot is less than 2 mm and each plot is about 2.5–4 m^2^ (Supplementary Fig. 4, Supplementary Fig. 5). Everyone photoed in Supplementary Fig. 4 and Supplementary Fig. 5 has allowed publication with human face. Though it is still unknown how much time is represented in the sampling plots and thus the effect of time averaging, we have confidence that all collected fossil individuals in a sampling layer do not represent long periods of elapsed time because intense volcanism suddenly killed and rapidly buried the organisms, resulting in thanatocoenoses^39^. Within the sampling layer, environmental conditions evidently changed minimally. All animal fossils were collected and identified by taxonomic specialists regardless of completeness or size of the identified specimens. We use OTUs (Operational Taxonomic Units) at all levels instead of scientific names. An operational Taxonomic Unit (OTU), typically at the species level, represents a taxon that is known and morphologically characterized but not yet established as a Linnean binomial. Consequently, use of this term facilitates analysis in ecological research prior to formal taxonomic description of the taxon in question. Using OTU is a more annotative and convenient method, such as the taxon Orthophlebiidae gen. sp1. New genera and species discovered during the experimental excavation were recorded, respectively, as gen1. nov. and sp1. nov. These new species will be described separately by the taxonomists participating in the study. Those species that could not be identified are recorded as sp1., sp2., etc. Some taxa that involve immatures and adults of the same species have large differences in values of indices from their occupied habitats and morphological functions. We marked a (l) after the scientific name to distinguish immatures (naiads, or larvae) from adults. Of all the collected materials, those sampled from Donggou are stored in the Inner Mongolia Museum of Natural History at Hohhot (IMMNH, Ai Kang, Collection Manager), China. All other specimens are housed in the fossil collection of the Key Lab of Insect Evolution and Environmental Changes, at the College of Life Sciences, Capital Normal University (CNUB; Dong Ren, curator), in Beijing, China.

Palaeoecological research with vertical sampling deviation on the scale of millimeters is rare. Importantly, we note that most palaeoecological datasets are obtained from float or talus specimens not linked to a known stratal interval, or from sampling from strata that is several or more centimeters thick^15^. For most non-catastrophic depositional environments, samples from strata several centimeters thick might represent a span of thousands of years^5^. Nevertheless, we infer that a sample set is suitable for network restoration of a fossil animal community, provided that the depositional environment does not exhibit drastic environmental changes within the vertical interval of the sampling layer (Supplementary Fig. 3).

### Companion software

We developed *TaphonomeAnalyst* (Fig. 1), an application program developed in Python, that allows researchers to easily perform exploratory analyses on common abundance profiles from taphocoenosis data. Although some analytical methods in our program can be found in many off-the-shelf microbiome analysis software^54^, such software cannot be fully adapted for the study of taphocoenoses. Compared with common visualization applications, such as Cytoscape and Gephi, *TaphonomeAnalyst* can provide statistical charts and network webs of fossil assemblages that conveniently support palaeoecological restoration from taphonomic data.

*TaphonomeAnalyst* is divided into six sections, each with a set of algorithms and procedures for implementation (Fig. 1). *TaphonomeAnalyst* is a user-friendly tool that integrates various sources of data, statistical methods, and visualization tools, furthermore, the *TaphonomeAnalyst* can provide a comprehensive analysis of a taphocoenosis, serve as a guide for fossil excavations, and display highly realistic networks of an ancient faunal community.

The key advantages of the *TaphonomeAnalyst* are the following.

1. The creation of a procedure to study fossil communities. By our sampling method, ancient faunal co-occurrence networks can be established that often are highly similar to that of extant communities.
2. It provides a powerful, convenient visual representation of the interaction network of a taphocoenosis.
3. The data input method is simple, which only requires fossil sample data from spreadsheets, with all functions that are easily realized by a step-by-step selection from a user menu.

### Evaluation of sampling effort and theoretical maximum biodiversity estimation (Module I)

This module provides the accepted standard for sampling coverage that is closely tied to the characteristics of the taphocoenosis^55,56^. Such a standard of sampling coverage should be focused not only to standing diversity, but also to potential diversity. Especially for the study of lacustrine faunas, the abundance of aquatic dominant species may be several orders of magnitude different from that of the terrestrial species. By contrast, terrestrial species may have more diversity but rare in relative abundance. Consequently, it is necessary to be attentive to changes in the potential diversity curve. Our software module not only provides *S*_*obs*_ diversity curves, but also provides diversity curves from ACE and Chao1 diversity estimators^57,58^.

$${{{{{\rm{C}}}}}}{{{{{\rm{h}}}}}}{{{{{\rm{ao}}}}}}1={S}_{{obs}}+\frac{{F}_{1}\left({F}_{1}-1\right)}{2{F}_{2}}$$

whereby ${S}_{{obs}}$: the direct observation diversity in the community. ${F}_{1}$: the number of OTUs containing only one individual. ${F}_{2}$: the number of OTUs containing only 2 individuals.

$${S}_{{ace}}={S}_{{abund}}+\frac{{S}_{{rare}}}{{C}_{{ace}}}+\frac{{F}_{1}}{{C}_{{ace}}}{\gamma }_{{ace}}^{2}$$

whereby ${{{{{S}}}}}_{{{{{abund}}}}}$: the number of abundant OTUs (with more than rare threshold individuals. Usually, the threshold is chosen as 10. But in our experience, a rare species threshold of 10 for paleoecology may be too large. Our software allows the user to set the rare species threshold.) when all samples are pooled^55–58^. ${S}_{{rare}}$: the number of rare OTUs (with less than or equal to rare threshold individuals) when all samples are pooled. ${{{{{C}}}}}_{{{{{ace}}}}}$: the sample abundance coverage estimator, ${F}_{1}$: the frequency of singletons. ${\gamma }_{{ace}}^{2}$: the estimated coefficient of variation for rare OTUs, is.

$${{{{{{\rm{\gamma }}}}}}}_{{{{{{\rm{ace}}}}}}}^{2}=\max \left[\frac{{S}_{{rare}}}{{C}_{{ace}}}\frac{\mathop{\sum }\nolimits_{i=1}^{10}i(i-1){F}_{I}}{({N}_{{rare}})({N}_{{rare}-1})}-1,0\right]$$

${S}_{{obs}}$ is the direct observational diversity, suited to evaluate the coverage of sampling in strata where aquatic species do not have a significant advantage. During the sampling process, some Daohugou samples could contain over 2000 *T. haifanggouensi* among 3000 individuals. In contrast, many terrestrial OTUs only have a few individuals. The *S*_*obs*_ curve may tend to flatten out when the number of samples is few, but sample location may still have significant potential diversity. Chao1 is sensitive to OTUs of only one individual, making it more suitable for plots where aquatic species dominate. The abundance coverage estimator considers a wider range of rare species and makes corrections for the coefficient of variation and sample coverage, which is more reasonable. However, due to the difference between the buried community and the present-day community, the definition of the abundance of rare species needs to be considered.

### Proportion of relative abundance of OTU analysis (Module II)

This module can provide bar graphs that display abundances. It should be emphasized that the abundance of species does not represent the abundance of species in the actual fossil community. Therefore, the relative abundance of an OTU is a poor indicator^28^. The abundance of species in a taphocoenosis is controlled by factors such as taxonomic group and size. To a certain extent, species abundances have the potential to reflect on the differing originating distances and trophic levels of the species within the fossil community.

### Proportion of taphonomic preservational grade of species analysis (Module III)

The taphonomic grade module defines the preservational quality of the fossils, as evidenced by their structural integrity, such as the degree of remaining articulations of joints displayed by the fossil (Fig. 5, Supplementary Fig. 4)^28,50,54,59,60^. Fossil taphonomic grade also can be used to explain the distance of the original organism’s habitat to its eventual deposition into lacustrine sediment. Though influenced by factors such as the robustness or gracileness of body parts, especially appendages, and body size, this method has been widely used in taphonomic analyses. This section provides taphonomic grade bar graphs for such taxa.

### Taphonomic environment analysis (Module IV)

This module allows for the output of hierarchical clusters, Venn diagrams, and heat maps. Hierarchical clustering uses Average assigns (clustering methods) and Bray-Curtis distance that are common methods in biodiversity research^61^. Hierarchical clustering is based on the abundance of aquatic OTUs in different plots (Filter condition is OTUs with a number of individuals greater than five) and can be used to divide sample sets into different groups from different taphonomic environments^61^. The software allows users to customize a list of aquatic OTUs. Venn diagrams show diversity differences within different taphonomic environments. Based on species abundance information, *TaphonomeAnalyst* can provide heat maps to conveniently discover gradients of species distributions as plots (Fig. 6a, b).

An Average assign is given by

$${{{{{{\bf{d}}}}}}}_{({{{{{\bf{u}}}}}},{{{{{\bf{v}}}}}})}=\mathop{\sum}\nolimits_{{{{{{\bf{ij}}}}}}}\frac{{{{{{\bf{d}}}}}}({{{{{\bf{u}}}}}}[{{{{{\bf{i}}}}}}],{{{{{\bf{v}}}}}}[{{{{{\bf{j}}}}}}])}{\left(\left|{{{{{\bf{u}}}}}}\right|* \left|{{{{{\bf{v}}}}}}\right|\right)}.$$

And the Bray-Curtis distance is given by

$${{{{{{\rm{d}}}}}}}_{({{{{{\rm{u}}}}}},{{{{{\rm{v}}}}}})}=\frac{\mathop{\sum}\nolimits_{{{{{{\rm{i}}}}}}}\left|{{{{{{\rm{u}}}}}}}_{{{{{{\rm{i}}}}}}}-{{{{{{\rm{v}}}}}}}_{{{{{{\rm{i}}}}}}}\right|}{\mathop{\sum}\nolimits_{{{{{{\rm{i}}}}}}}\left|{{{{{{\rm{u}}}}}}}_{{{{{{\rm{i}}}}}}}+{{{{{{\rm{v}}}}}}}_{{{{{{\rm{i}}}}}}}\right|}.$$

### Species correlation analysis (Module V)

The purpose of the species correlation analysis module is to discover potential interactions among fossil species, such that these interactions reflect symbiotic relationships. *TaphonomeAnalyst* provides several simple methods for calculating correlations and outputting semi-matrix figures, such as Pearson’s product-moment correlation, and Spearman’s and Kendall’s rank correlation coefficients (Supplementary Fig. 4)^62,63^.

### Correlational network visualization (Module V)

A network with links has a combination of nodes and edges. In such a network, nodes represent taxa that occur in sampling plots. The edges of the network are taphonomic co-occurrences between taxa. During the deposition process, biological remains exist in the same subenvironment that add to the taphonomic process. Therefore, taphonomic co-occurrence can be explained by the overlapping of habitats between two taxa to a certain extent. The correlational network visualization module uses the Louvain algorithm to provide clusters within the network that allows users to set up their own filters, such as correlation strengths and *P* values^64^. The Louvain algorithm is a method that discovers communities based on modularity, which performs well in efficiency and effectiveness, and aims to discover the structure of hierarchical modules. Its goal is to optimize and maximize the modularity of the entire graph attribute structure, or community network. The resulting clustering effect is particularly evident for graphs with fewer edges^64^.

### Daohugou animal network based on morphologic function and taxonomic uniformitarianism

We aggregate morphologic function and taxonomic uniformitarian data for all families present in the sampling plots (Supplementary table 1). We also visualize networks in *Python* using functions from the networks and matplotlib libraries.

### Reporting summary

Further information on research design is available in the Nature Portfolio Reporting Summary linked to this article.

### Supplementary information

Supplementary information

Description of Supplementary Materials

Reporting Summary

Supplementary data 1

## Data Availability

Supplementary Data 1 is provided with the paper compiling source data behind all graphs and tables shown.

## Code availability

The code of *TaphonomeAnalyst* can be downloaded from https://github.com/wma1995/TaphonomeAnalyst and https://zenodo.org/record/8333176 .
