# Supplementary material for: A new method for examining the co-occurrence network of fossil assemblages
Source: Commun Biol. 2023 Oct 31;6:1102. doi: 10.1038/s42003-023-05417-6 (PMC10618518; doi:10.1038/s42003-023-05417-6)
Supplement: Supplementary file 1 — Supplementary information [file 42003_2023_5417_MOESM1_ESM.pdf]

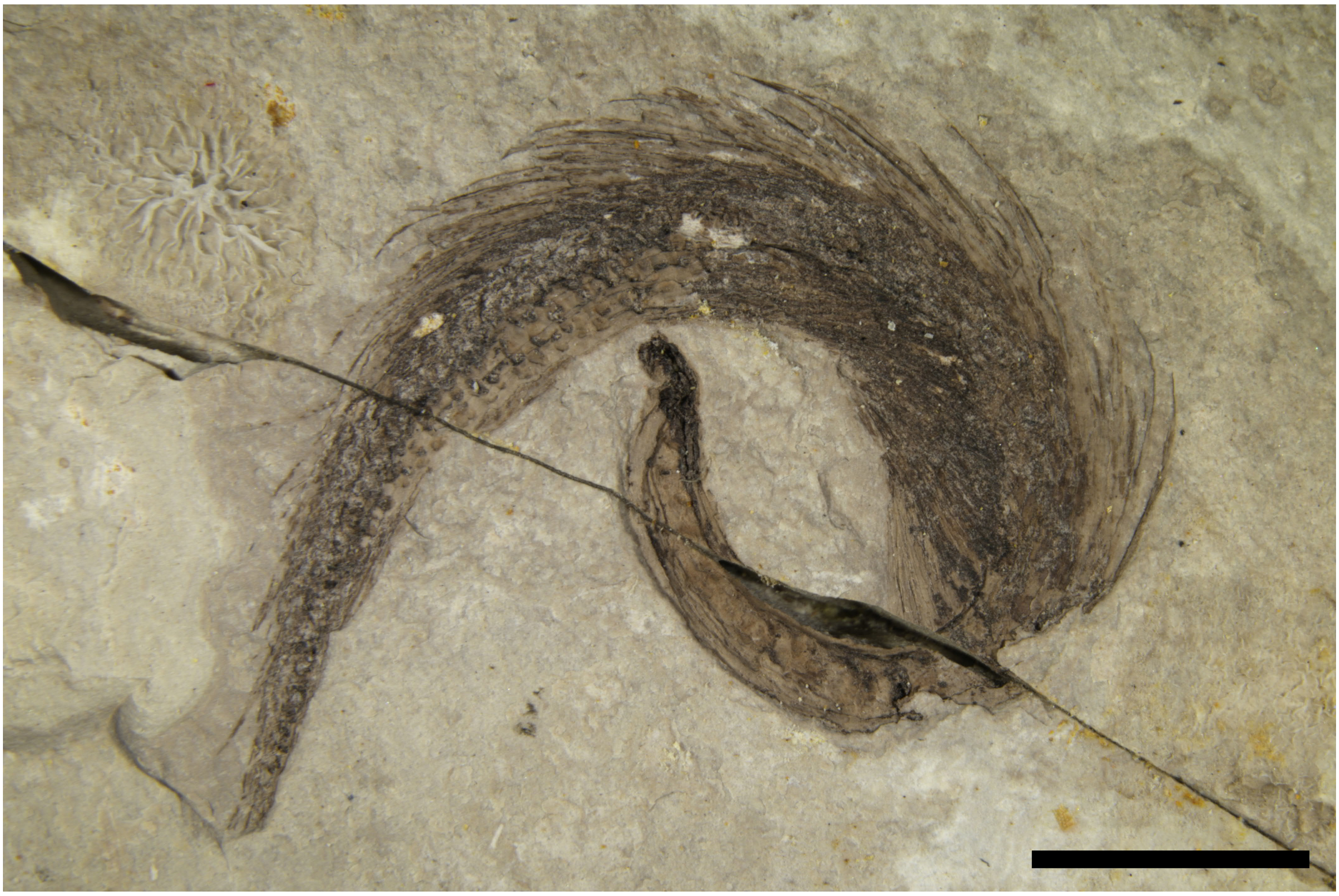

Supplementary Figure 1 Lamprey fossil found in Donggou. Scale bar: 5 mm.

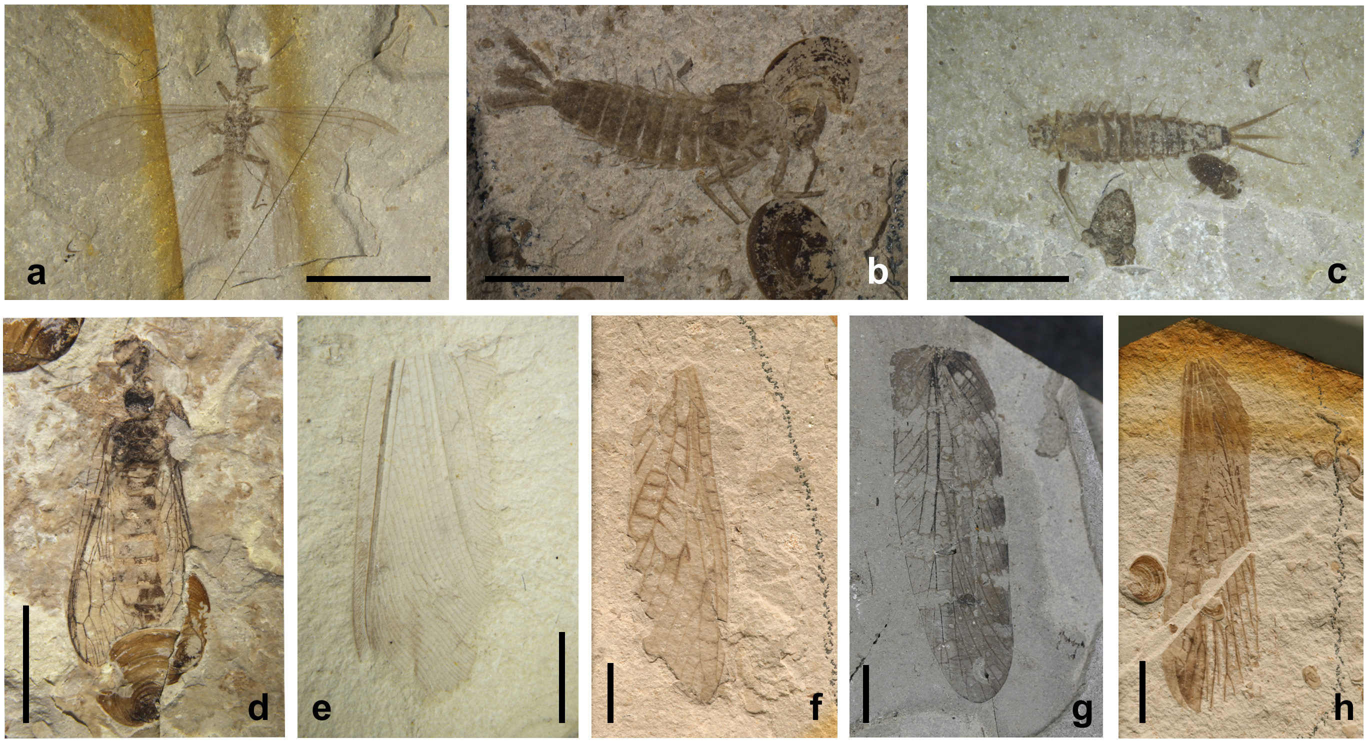

Supplementary Figure 2 Taxa considered to be alpine organisms. (**a**) *Pronemoura* sp. (Pronemouridae). (**b**) *Shantous lacustris* (Hexagenitidae). **(c)** *Mesobaetis sibirica* (Siphlonuridae). (**d**) Mesoraphidiidae. (**e**) Osmylidae. (**f**) Pteronarcyidae. (**g**) Prophalangopsidae. (**h)** Bajanzhargalanidae. All scale bars: 5 mm. This photographic plate was photoed and created by Yunyu Tang (Co-first-author of this article). All elements used in this plate are first published.

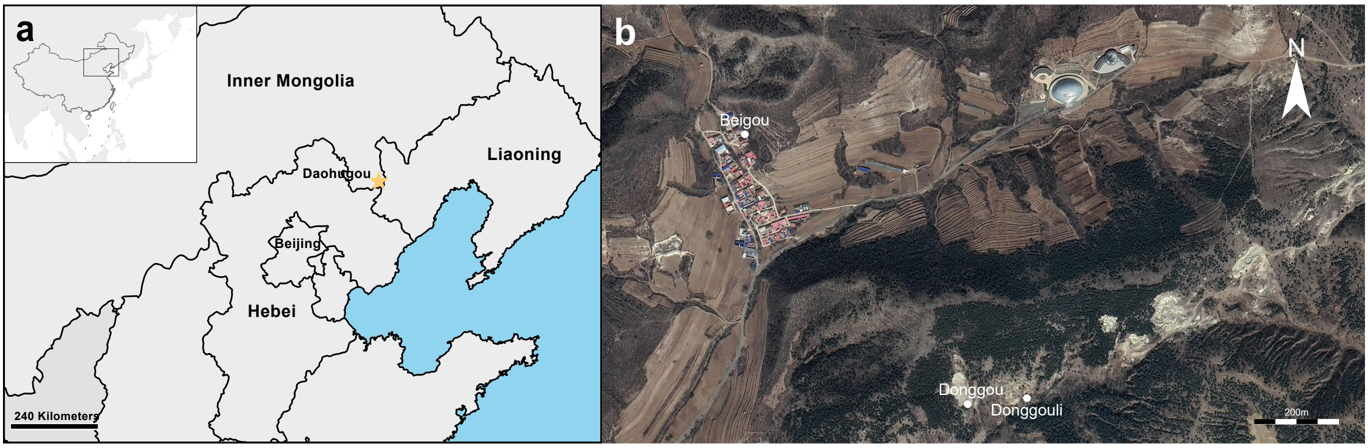

Supplementary Figure 3 Geographic setting of the sampling localities. a, The location of Daohugou Valley in China, mapped by ArcGIS. **b**, The Donggouli plots are located at 41°18'49.42"N , 11913'50.67"E. TheDonggou plots are located at 41°18'48.95"N, 119°1344.58"E. The Beigou plots are located at 41°19'9.77"N,11913'21.46"E, These locations were plotted using Google Map.

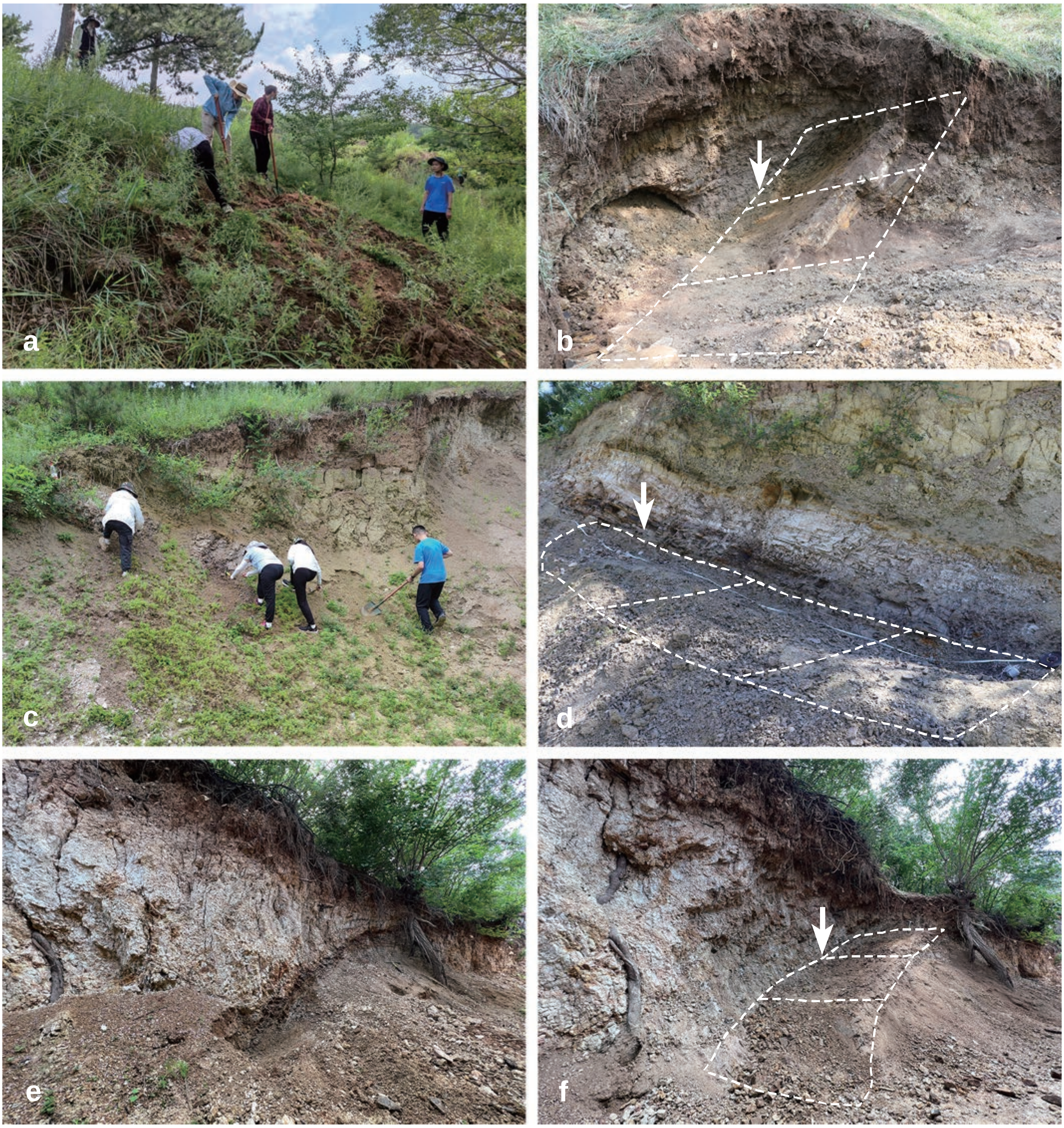

Supplementary Figure 4 Field work. a, **b**, Donggouli plots; **c**, **d**, Donggou plots; **e**, **f**, Beigou plots.

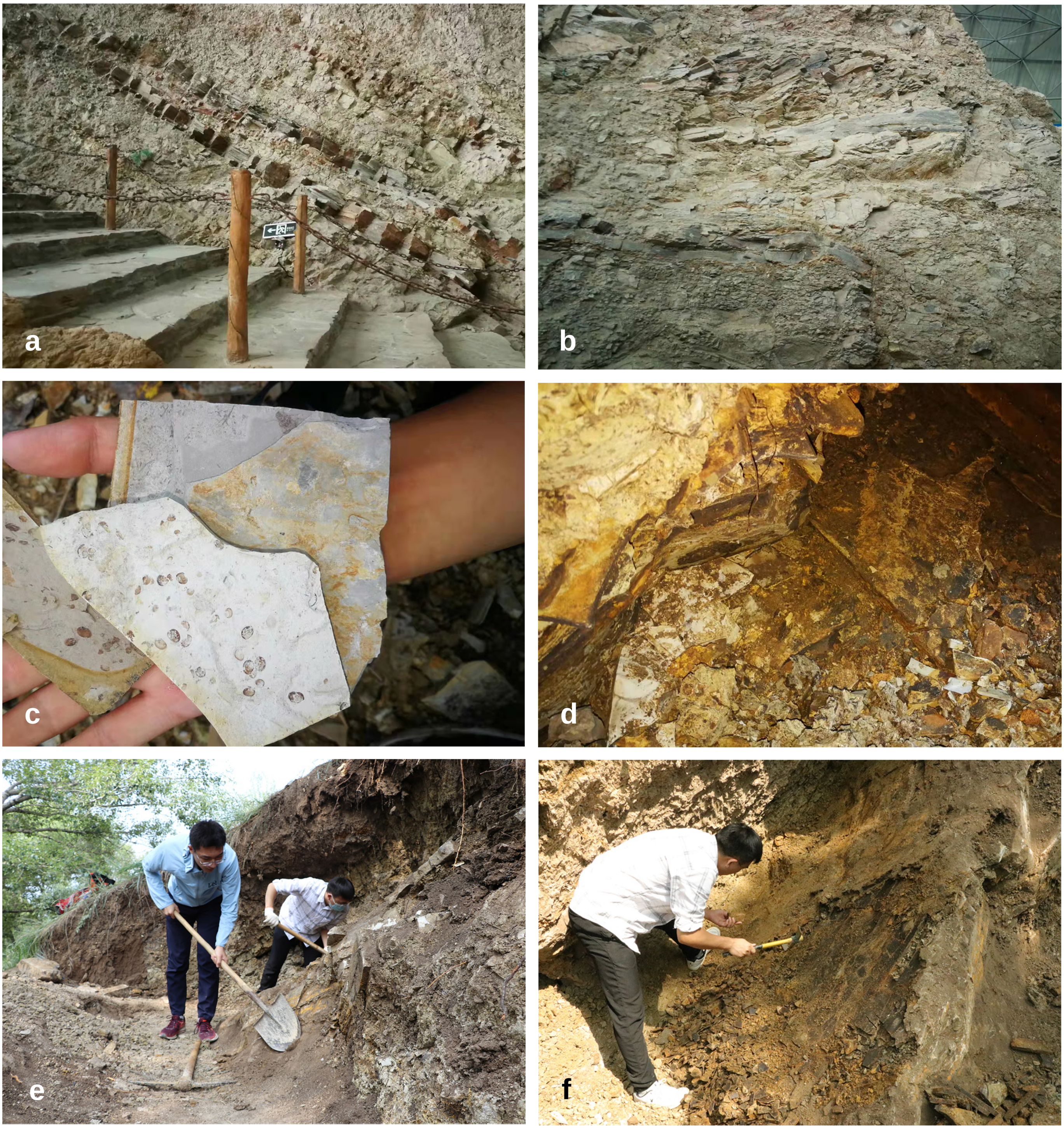

Supplementary Figure 5 Sampling lithology and process. a, **b**, Lacustrine sedimentary rhythmites from a standard section in the Daohugou Museum. **c**, Three lithological fossils layers. **d**, **e**, **f**, Images of the sampling process

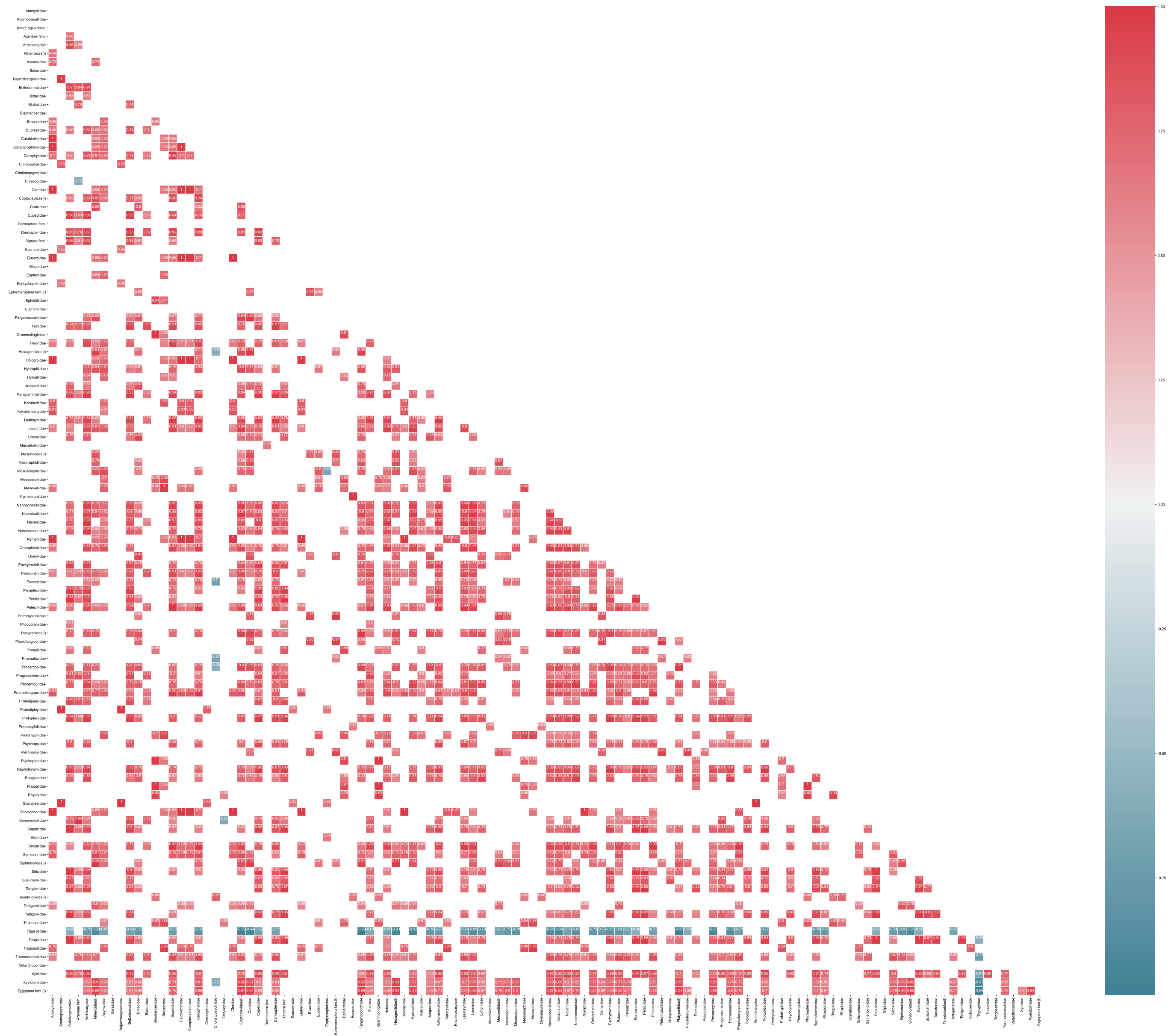

Supplementary Figure 6 Heat map of family-level OTU correlation. The filter condition is P < 0.1. Red colors are positive correlations; blue colors are negative correlations.

Supplementary Table 1 The standard of taphonomic grades.

Taphonomic grades	Preservation		
	**Clam shrimps and bivalves**	**Other arthropods**	**Vertebrates**
A	Shell edge > 90% preserved. growth bands are fully clear.	> 90% preserved. Body articulated, wing veins visible and almost complete.	Body and limbs are complete and articulated.
B	Shell edge > 70% preserved. growth bands are almost clear.	80–90% preserved. Body almost complete, including head, thorax, abdomen and thoracic appendages, details such as antennae or cerci lost.	70–80% torso and limbs are complete. Partial joint displacement.
C	Shell edge > 60% preserved. growth bands are partially clear.	60–80% preserved. Body deformed, at least one of six legs lost.	60–70% torso preserved.
D	Shell edge >50% preserved.	30–60% preserved. Wings disarticulated, remains of head, thorax and abdomen preserved.	Torso with missing tail or head.
E	Shell fragments.	< 30% preserved. High disarticulated body, isolated structures such as single legs, abdomen and/or wings preserved.	Scattered bones.
