# Supplementary material for: A new method for examining the co-occurrence network of fossil assemblages
Source: Commun Biol. 2023 Oct 31;6:1102. doi: 10.1038/s42003-023-05417-6 (PMC10618518; doi:10.1038/s42003-023-05417-6)
Supplement: Supplementary file 3 — Reporting Summary [file 42003_2023_5417_MOESM3_ESM.pdf]

nature portfolio

Corresponding author(s): Dong Ren

Last updated by author(s): Sep 12, 2023

Reporting Summary

Nature Portfolio wishes to improve the reproducibility of the work that we publish. This form provides structure for consistency and transparency in reporting. For further information on Nature Portfolio policies, see our Editorial Policies and the Editorial Policy Checklist.

Statistics

For all statistical analyses, confirm that the following items are present in the figure legend, table legend, main text, or Methods section.

n/a	Confirmed	
☐	☒	The exact sample size (n) for each experimental group/condition, given as a discrete number and unit of measurement
☐	☒	A statement on whether measurements were taken from distinct samples or whether the same sample was measured repeatedly
☒	☐	The statistical test(s) used AND whether they are one- or two-sided<br>*Only common tests should be described solely by name; describe more complex techniques in the Methods section.*
☐	☐	A description of all covariates tested
☐	☐	A description of any assumptions or corrections, such as tests of normality and adjustment for multiple comparisons
☐	☐	A full description of the statistical parameters including central tendency (e.g. means) or other basic estimates (e.g. regression coefficient) AND variation (e.g. standard deviation) or associated estimates of uncertainty (e.g. confidence intervals)
☐	☐	For null hypothesis testing, the test statistic (e.g. F, t, r) with confidence intervals, effect sizes, degrees of freedom and P value noted<br>*Give P values as exact values whenever suitable.*
☒	☐	For Bayesian analysis, information on the choice of priors and Markov chain Monte Carlo settings
☐	☒	For hierarchical and complex designs, identification of the appropriate level for tests and full reporting of outcomes
☐	☒	Estimates of effect sizes (e.g. Cohen's d, Pearson's r), indicating how they were calculated

Our web collection on statistics for biologists contains articles on many of the points above.

Software and code

Policy information about availability of computer code

Data collection	The data comes from the abundance of fossils in the excavation plots.
Data analysis	The code of TaphonomeAnalyst can be downloaded from https://github.com/wma1995/TaphonomeAnalyst, which we provide in the latest version of he manuscript.

For manuscripts utilizing custom algorithms or software that are central to the research but not yet described in published literature, software must be made available to editors and reviewers. We strongly encourage code deposition in a community repository (e.g. GitHub). See the Nature Portfolio guidelines for submitting code & software for further information.

Data

Policy information about availability of data

All manuscripts must include a data availability statement. This statement should provide the following information, where applicable:

- Accession codes, unique identifiers, or web links for publicly available datasets
- A description of any restrictions on data availability
- For clinical datasets or third party data, please ensure that the statement adheres to our policy

The code of TaphonomeAnalyst can be downloaded from https://github.com/wma1995/TaphonomeAnalyst, which we provide in the latest version of the manuscript.

Research involving human participants, their data, or biological material

Policy information about studies with human participants or human data. See also policy information about sex, gender (identity/presentation), and sexual orientation and race, ethnicity and racism.

Reporting on sex and gender	Paleontological research does not involve this item.
Reporting on race, ethnicity, or other socially relevant groupings	Paleontological research does not involve this item.
Population characteristics	Paleontological research does not involve this item.
Recruitment	Paleontological research does not involve this item.
Ethics oversight	Paleontological research does not involve this item.

Note that full information on the approval of the study protocol must also be provided in the manuscript.

Field-specific reporting

Please select the one below that is the best fit for your research. If you are not sure, read the appropriate sections before making your selection.

☐ Life sciences ☐ Behavioural & social sciences ☒ Ecological, evolutionary & environmental sciences

For a reference copy of the document with all sections, see nature.com/documents/nr-reporting-summary-flat.pdf

Ecological, evolutionary & environmental sciences study design

All studies must disclose on these points even when the disclosure is negative.

Study description	We developed a software, the TaphonomeAnalyst, to study the associational network of lacustrine entombed fauna, or taphocoenosis. TaphonomeAnalyst allows researchers to easily perform exploratory analyses on common abundance profiles from taphocoenosis data. The dataset for these investigations resulted from fieldwork of the latest Middle Jurassic Jiulongshan Formation near Daohugou Village, in Ningcheng County of Inner Mongolia, China, spotlighting the core assemblage of the Yanliao Fauna.
Research sample	Our fossil sample sets were collected from the latest Middle Jurassic Jiulongshan Formation at Daohugou Village, in Ningcheng County of Inner Mongolia, China.
Sampling strategy	We selected three fossil locations for excavation based on lithology. The center of each sampling plot was separated 2 m apart from an adjacent sampling plot The lithologies of all strata from the three locations consist of well-bedded, tuffaceous shale.
Data collection	The data comes from the abundance of fossils in the excavation plots.
Timing and spatial scale	2-4 square meters per for plots.
Data exclusions	None.
Reproducibility	Based on unpublished data from other regions, we believe it is reproducible.
Randomization	None.
Blinding	None.

Did the study involve field work? ☒ Yes ☐ No

Field work, collection and transport

Field conditions	Based on the stratigraphic inter fingering of a lacustrine and a volcaniclastic apron facies, the sedimentary environment of Jiulongshan Formation is interpreted as several fluctuating, shallow lakes with distant volcanogenic sedimentary input. Radiometric evidence shows that the depositional age of the Jiulongshan Formation is 165–164 Ma and is time equivalent to the Haifanggou Formation in Liaoning Province.
Location	Our fossil sample sets were collected from the latest Middle Jurassic Jiulongshan Formation at Daohugou Village, in Ningcheng County of Inner Mongolia, China.

Access & import/export	This study does not involve the entry and exit of materials
Disturbance	None.

Reporting for specific materials, systems and methods

We require information from authors about some types of materials, experimental systems and methods used in many studies. Here, indicate whether each material, system or method listed is relevant to your study. If you are not sure if a list item applies to your research, read the appropriate section before selecting a response.

Materials & experimental systems

n/a	Involved in the study
☒	☐ Antibodies
☒	☐ Eukaryotic cell lines
☐	☒ Palaeontology and archaeology
☒	☐ Animals and other organisms
☒	☐ Clinical data
☒	☐ Dual use research of concern
☒	☐ Plants

Methods

n/a	Involved in the study
☒	☐ ChIP-seq
☒	☐ Flow cytometry
☒	☐ MRI-based neuroimaging

Palaeontology and Archaeology

Specimen provenance	Ningcheng National Geopark in Inner Mongolia was designated as the teaching and scientific research practice base of Capital Normal University in 2020. (Approval document is Capital Normal University School Letter [2020] No. 10 Issued by: Meng Fanhua).
Specimen deposition	Based on the stratigraphic inter fingering of a lacustrine and a volcaniclastic apron facies, the sedimentary environment of Jiulongshan Formation is interpreted as several fluctuating, shallow lakes with distant volcanogenic sedimentary input. Radiometric evidence shows that the depositional age of the Jiulongshan Formation is 165–164 Ma and is time equivalent to the Haifanggou Formation in Liaoning Province.
Dating methods	Radiometric evidence shows that the depositional age of the Jiulongshan Formation is 165–164 Ma and is time equivalent to the Haifanggou Formation in Liaoning Province.

☒ Tick this box to confirm that the raw and calibrated dates are available in the paper or in Supplementary Information.

Ethics oversight	Fossil collection is conducted sporadically, in accordance with relevant national regulations.

Note that full information on the approval of the study protocol must also be provided in the manuscript.
